# Supplementary material for: Synovial Fluid Interleukin Levels Cannot Distinguish between Prosthetic Joint Infection and Active Rheumatoid Arthritis after Hip or Knee Arthroplasty
Source: Diagnostics (Basel). 2022 May 11;12(5):1196. doi: 10.3390/diagnostics12051196 (PMC9140440; doi:10.3390/diagnostics12051196)
Supplement: Supplementary file 1 [file diagnostics-12-01196-s001.zip › diagnostics-1694582-supplementary.pdf]

Supplement Figure S1. Scatterplots showing the concentration of ESR and CRP in the three groups. *p-value < 0.05; **p-value < 0.01; *** p-value< 0.001; **** p-value< 0.0001

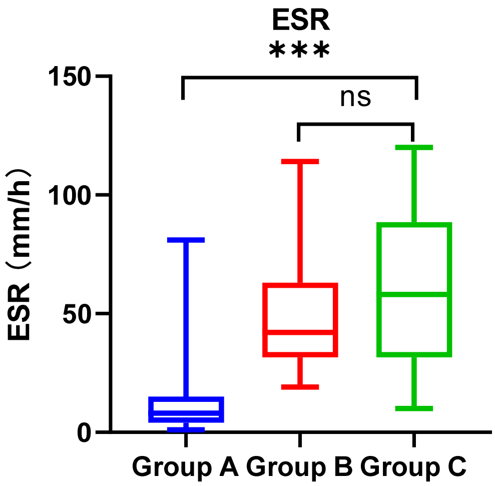

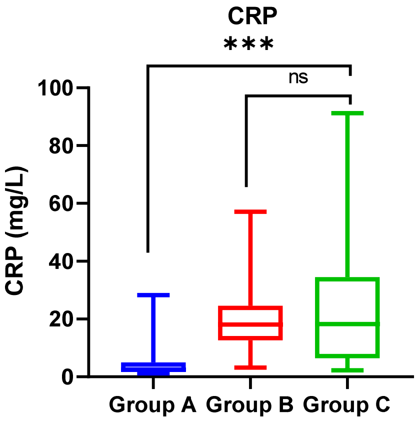

Supplement Table S1. Receiver operating characteristic analysis of ESR and CRP level between the PJI and aseptic loosening groups.

Markers	Cut-off value	AUC (95%CI）	Sensitivity (95%CI）	Specificity (95%CI）
ESR (mm/h)	40	0.719 (0.6912 to 0.8427)	73.64 (59.46% to 86.12%)	71.15 (57.47% to 82.27%)
CRP (mg/L)	11.3	0.781(0.6710 to 0.8450)	81.13 (68.30% to 90.16%)	67.67 (53.31% to 82.08%)

CRP, C-reactive protein; ESR, erythrocyte sedimentation rate, CI, confidence interval
